# 3PFDB+: improved search protocol and update for the identification of representatives of protein sequence domain families

**DOI:** 10.1093/database/bau026

**Published:** 2014-04-03

**Authors:** Agnel P. Joseph, Prashant Shingate, Atul K. Upadhyay, R. Sowdhamini

**Affiliations:** ^1^National Centre for Biological Sciences (TIFR), GKVK Campus, Bellary Road, Bangalore 560065, Karnataka, India.; ^2^Manipal University, Madhav Nagar, Manipal 576104, Karnataka, India.

## Abstract

Protein domain families are usually classified on the basis of similarity of amino acid sequences. Selection of a single representative sequence for each family provides targets for structure determination or modeling and also enables fast sequence searches to associate new members to a family. Such a selection could be challenging since some of these domain families exhibit huge variation depending on the number of members in the family, the average family sequence length or the extent of sequence divergence within a family. We had earlier created 3PFDB database as a repository of best representative sequences, selected from each PFAM domain family on the basis of high coverage. In this study, we have improved the database using more efficient strategies for the initial generation of sequence profiles and implement two independent methods, FASSM and HMMER, for identifying family members. HMMER employs a global sequence similarity search, while FASSM relies on motif identification and matching. This improved and updated database, 3PFDB+ generated in this study, provides representative sequences and profiles for PFAM families, with 13 519 family representatives having more than 90% family coverage. The representative sequence is also highlighted in a two-dimensional plot, which reflects the relative divergence between family members. Representatives belonging to small families with short sequences are mainly associated with low coverage. The set of sequences not recognized by the family representative profiles, highlight several potential false or weak family associations in PFAM. Partial domains and fragments dominate such cases, along with sequences that are highly diverged or different from other family members. Some of these outliers were also predicted to have different secondary structure contents, which reflect different putative structure or functional roles for these domain sequences.

**Database URL:** http://caps.ncbs.res.in/3pfdbplus/

## Introduction

Proteins are one of the fundamental biomolecules important for cellular integrity and survival. They perform many important and varying roles like catalysing reactions, signal-transmission, transporting different molecules or ions, stabilizing cytoskeleton, *etc*. Proteins sharing similar sequences tend to have similar structures and subsequently similar functions (1). These related proteins can be classified at sequence, structural and functional levels. In this high throughput sequencing era, determination of structure and function of proteins cannot match the rate of incoming sequence information. This results in a large portion of sequenced data being with no structural information or with no function annotation. Hence, the use of different computational approaches has become inevitable for obtaining structural or functional insights.

Proteins are often described to have compact structural or functional units called domains. These domains exhibit significant conservation with respect to both amino acid sequence and tertiary fold (2). Assignment of domains to protein sequences or structures facilitate classification and functional annotation. PFAM (3) database is an excellent attempt to facilitate the function annotation based on domain assignments. This database consists of protein domain families which are automatically classified on the basis of sequence similarities, built around Hidden Markov Models (HMM). A HMM profile derived from a set of representative seed sequences, is associated to each domain family. New sequences are grouped under one family based on comparison with the family HMM profile. Hence, the family seed dataset reflects multiple representatives covering all the sequence information of the family.

The composition and size of PFAM families vary significantly. In some families, members are very similar to each other, while in other cases, sequence identity between members is quite low. On an average, the seed sets are seen to span a sequence identity range of 30–40%. At this sequence identity range and especially in case of highly diverse families, multiple sequence alignments may not be trivial. This may deteriorate the quality of HMM profiles generated for these families. Profiles generated with sequences in the identity range between 30% and 50% are reported to be efficient in homology detection (4). Besides, the sequence divergence within a family, the average sequence length also varies from 6 (PF08261) to 1402 (PF06317) residues. Short proteins may falsely associate with a part of HMM model of certain families. PFAM family assignments could have ambiguities when the proteins have signal peptides or transmembrane helices (5). Such wrong family assignments might reduce reliability of the classification and the selection of representative seed dataset.

3PFDB database was designed to find a best representative sequence (BRS) for each PFAM family (6). A profile generated from the BRS, (best representative profile, BRP) is expected to identify maximum number of family members. These profiles can provide a more refined representation, owing to the large diversity observed in certain families. Sensitive homology detection methods like HMMER (7), FASSM (8) and RPS-BLAST (9, 10) were used for associate new sequences with the representative family profiles. Repertoire of representative family sequences can be used to carry out simple sequence searches which are computationally very fast and they also serve as targets for structure determination or computational modeling.

In PFAM database, whole domain alignments are used for constructing models without any added information on the conserved motifs. Small conserved motifs often reflect family signatures. One of our in-house programs, FASSM (8) (Function Association using Sequence and Structural Motifs) can detect remote homologs evolved through circular permutation or discontinuous domains. This algorithm is quite sensitive and can detect homologs for small proteins with few conserved residues.

In this work, we improve the 3PFDB database using more efficient strategies for generation of BRPs and implement the two methods FASSM (8) and HMMER3 (7) for identifying family members. RPS-BLAST (9) was not used in this study, due to the relatively poor performance when compared to these two methods. Further, instead of using PFAM seeds for generating representative profiles, independent sequence sets gathered with an identity threshold of 50%, were used. The framework of the current protocol permits easier automation and periodic updates of the database.

The members which failed to recognize their family representative profiles were further assessed to check the reliability of their family association. These sequences could not be associated with the family profile by both motif (FASSM) and sequence based (HMMER) approaches. Our assumption was that certain sequences in a family might have diverged extensively with respect to other family members. However, we observed several cases of weak or false associations in a family. Those families where the representatives give poor coverage are studied in detail in terms of sequence dispersion, average length and family size. Multiple representatives were selected for those families where a single BRS gives low coverage. The updated version of the database, 3PFDB+, provides these new sets of representative sequences and profiles. The dispersion of sequences in each family is represented with a PCA plot and the location of BRS is highlighted. Representative profiles are provided as HMM models and the motifs identified in each family are also given. Users can also search any sequence of interest against the representative profiles using HMMER (7) and FASSM (8).

## Methods

**Figure 1.**
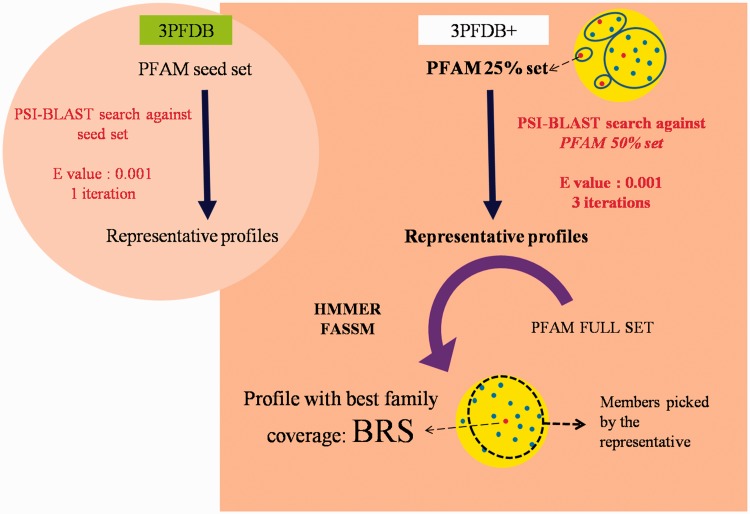
Workflow for the identification BRSs and associated profiles in 3PFDB+ database. The portion highlighted in the circular background shows the differences in the profile generation approach in the earlier study (6).

PFAM v26, consisting of 13 672 families, was used to identify representatives in each domain family. To extract the set of possible representatives, all the family members were clustered at a sequence identity threshold of 25% using BlastClust (11). From each cluster, the longest sequence was chosen, to form the seed dataset. Profiles corresponding to the representative sequences were generated by performing PSI-BLAST (12) searches against a non-redundant PFAM family dataset gathered at 50% identity cut-off (Figure 1). The searches were performed for three iterations at an E-value threshold of 10^–^^3^. In an earlier study, three different sequence similarity search methods were compared, for their efficiency in identifying representatives, for a small dataset of 100 PFAM families (10). The same dataset was used for optimizing the improved protocol devised in this work.

The set of sequences generated from PSI-BLAST (12) runs forms the representative profiles. Each of these profiles was then assessed for the family coverage using sensitive tools like HMMER (7) and FASSM (8). The efficiency in identifying other members from the same PFAM family was computed as the family coverage. In case of HMMER (7), an E-value cut-off of 10^–^^2^ was used to associate family members. FASSM (8) program incorporates different parameters, *viz.*, size of the motif, number of the motifs allowed in the family, order of the motifs, distance between motifs, motif conservation score, *etc*. FASSM (8) program employs neural network with optimized weights for each parameter to decide whether query sequence belongs to given PFAM family. The coverage for each seed sequence of PFAM family was calculated and that seed with maximum coverage were considered as BRS.

For sequences not associated with the representative profiles, detailed analysis was carried out. Low complexity regions were detected using SEG (13) with a minimal length of 10 residues and transmembrane proteins were predicted using SOSUI (14) and TM-HMM (15).

## Results

**Figure 2.**
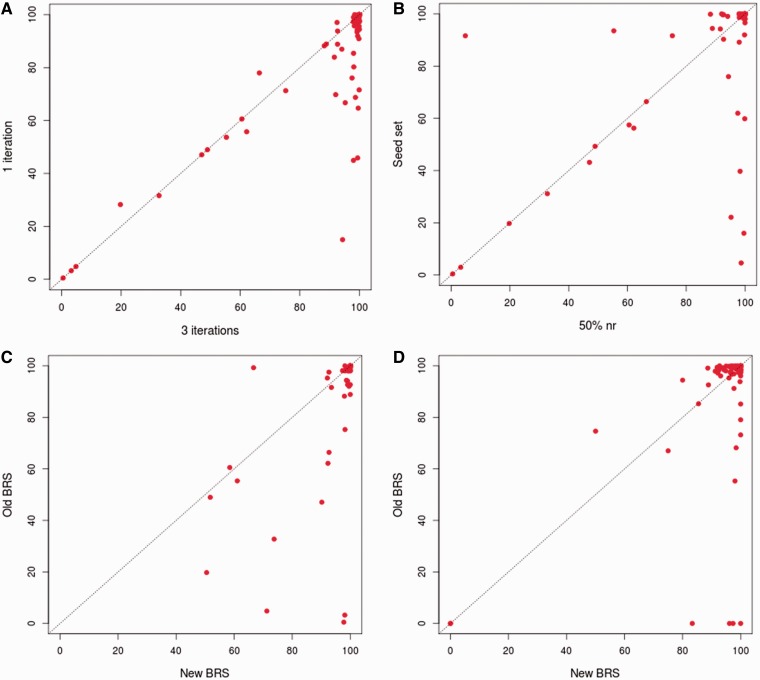
Comparison of representative coverage in a family. (**A**) Comparison of coverage obtained where the representative family profile is generated using three iterations of PSI-BLAST and the ones generated from single iteration. The PSI-BLAST (12) searches were carried out against the 50% non-redundant set of the family. The coverage was calculated by checking family associations using HMMER (7) (**B**) Comparison of coverage obtained when is the representative family profile is generated by performing three iterations of PSI-BLAST searches against the 50% non-redundant set of the family and those obtained when the search is carried out against PFAM seed dataset (3). The coverage was calculated by checking family associations using HMMER. (**C**) Comparison of coverage obtained using the representatives derived based on 3PFDB+ protocol and those obtained with representatives identified in the earlier work (6). The coverage was calculated by checking family associations using HMMER. (**D**) Comparison of coverage obtained using the representatives derived based on 3PFDB+ protocol and those obtained with representatives identified in the earlier work (6). The coverage was calculated by checking family associations using FASSM (8).

The new approach, as followed in 3PFDB+, was tested on the 100-family dataset used for the earlier 3PFDB analysis (10). As expected, profiles generated from three PSI-BLAST (12) iterations provide better coverage in almost all families when compared to a simple BLAST search (Figure 2A). The choice of an initial dataset, at 50% non-redundancy cut-off, for generating profiles also improved the coverage when compared to the seed set (Figure 2B). Only in two families, coverage was quite higher (86% and 16% increase), with the original seed dataset proposed within PFAM. For both families, seed set had more sequences (start points) than the 50% non-redundant dataset. Hence, enriching these profiles with more sequence information can give better coverage in these cases.

### Improvement with new approach

For the 100-family dataset (10), new representative sequences were identified with the improved protocol (Figure 1). The family coverage of profiles derived from these representatives was then compared to that obtained with the previously identified representatives (6) (Figure 2C and D). Best representatives were selected separately using two efficient sequence homology detection methods, HMMER (7) and FASSM (8). In case of HMMER (7) derived representatives, 99% of them had similar or better coverage (Figure 2C). Only for one family, Picornavirus core protein 2A (PFAM ID: PF00947), the coverage was 32% lower with the use of new representative profile. The sequences in this family are highly conserved and the 50% non-redundant set was left with only four sequences, out of which three are partial domains (<60% of average family length). Hence the quality of representative profile was reduced and enrichment with more family sequences was required. FASSM-based representatives also showed significant improvement in coverage (Figure 2D). Only for two family representatives, Picornavirus core protein 2A and Rotavirus NS26 family, the coverage values were significantly lower than that obtained with representatives previously identified for these families.

In a small percent of families, representative profile coverage was low, and the 50%-set had only a few sequences. These profiles were enriched by carrying out searches against the full family set, instead of 50% non-redundant set. This was performed for all families where the best representative coverage was <90%. Profile enrichment resulted in improvement of coverage for about 644 (out of 1160) and 55 (out of 1605) families using HMMER (7) and FASSM (8), respectively.

### HMMER vs FASSM

**Figure 3.**
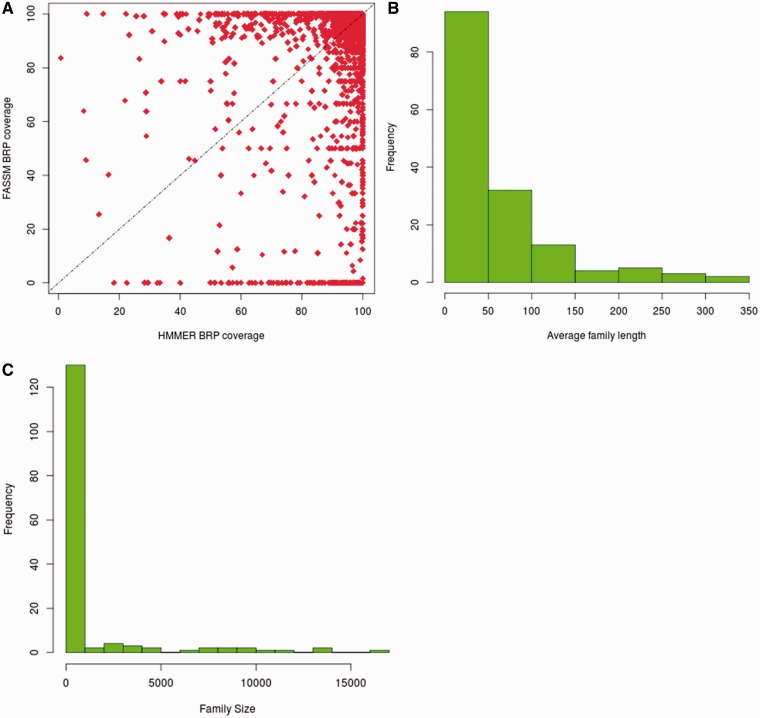
Coverage obtained with best representatives identified in 3PFDB+. (**A**) Comparison of family coverage of best representatives identified by HMMER (3) and FASSM (8). For the 153 families where the representatives by both methods had coverage <90%, (**B**) the distribution of average family sequence length and (**C**) family size, are plotted.

The BRSs, identified by HMMER and FASSM, were compared for their family coverage. In the case of HMMER, 13 214 representatives retain family coverage of more than 90%, while 12 122 representatives identified using FASSM exhibit coverage more than 90% (Figure 3A). For 3473 families, the same BRS were identified by both HMMER and FASSM. The coverage obtained for representatives, identified by HMMER, is better than that chosen using FASSM for 33.5% of families. However, FASSM-based representatives retained better coverage in only 16.6% of cases.

Only for 153 out of 13 672 families, the representatives identified by both methods had coverage <90%. Out of these families, 94 retained average family length <50 residues and 97 families had <100 members (Figure 3B and C). Hence, the families exhibiting low coverage are small families with short sequences. 73% of these families, where representatives had low coverage, do not belong to any PFAM clan. The clan covering 10 of these families is the tetratricopeptide repeat superfamily, whose members are involved in diverse cellular activities like cell cycle regulation, transcriptional control, protein transport, neurogenesis and folding (16). The underlying families involve short repeat sequences and are inherently highly diverse.

### Scope of the database

The updated database of PFAM family representatives covers 13 672 families from PFAM v 26 (3), and for 13 519 (∼99%) families, the representatives have more than 90% coverage. BRSs were identified using HMMER (7) and FASSM (8) which are highly efficient in detecting sequence homologs by global sequence similarity and local motif matching, respectively. Best representatives chosen by each of these methods are now provided. Users can also search new sequences against the dataset of best representatives to check the association with any PFAM family.

MySQL is used as a backend for 3PFDB+ database. All the CGI scripts for the server side are coded in PERL. FASSM scripts are coded in C++ and PERL. In this update, we have provided useful information about the PFAM protein families and user-friendly access options (see Supplementary Table S1 for details).

**Figure 4.**
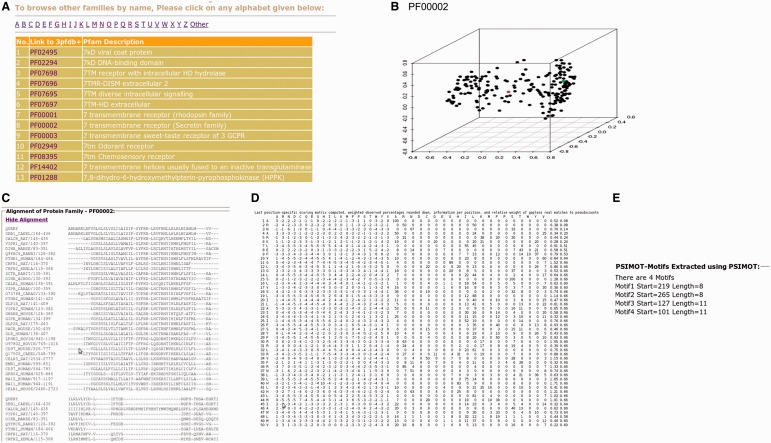
Some features of 3PFDB+ database. BRSs and profiles corresponding to each PFAM family can be searched from a (**A**) list of families. A (**B**) PCA plot highlights the sequence divergence in the family and also gives the location of the best representative (red). The BRP can be accessed as a (**C**) multiple sequence alignment, (**D**) PSSM or HMM model. (**E**) Family-specific sequence motifs identified by FASSM (8) are also presented.

The improved and updated version of 3PFDB database (3PFDB+) provides the new sets of representatives for all PFAM families (Figure 4). A 3D-PCA plot reflects the sequence divergence in the family and also highlights the location of the representative sequence among the members in the 50% non-redundant set. The conserved motifs identified in the family using FASSM are also listed in the database. User can also download the PSSMs, multiple sequence alignments and HMM models corresponding to the BRPs (Figure 4). The complete dataset of representative profiles can be downloaded from http://caps.ncbs.res.in/3pfdbplus/PFAM_BRP/.

### Comparison with PFAM domain assignment

In order to realize the importance of 3PFDB+ representatives in domain assignments in comparison to PFAM HMM profiles, we randomly selected a set of 50 reviewed human protein entries from UNIPROT where the domain assignments were different. These sequences were associated with PFAM HMM profiles and 3PFDB+ BRPs by carrying out HMMscan searches at E-value threshold of 10^–^^2^. For 29 sequences, different but related (same PFAM clan) domains were assigned by 3PFDB+ and PFAM. Loose family definitions and tendency of certain members to associate strongly with related families have been an important concern and family-specific gathering threshold scores were implemented to alleviate such cross-talks (17). However the quality of seed profile needs to be checked for underlying problems of ‘profile dilution’. In this analysis, we have not incorporated GA scores for PFAM searches as we aim to have a direct comparison of associations based on the profiles. 16 out of 29 related family associations, involves closely related domain sequences that were grouped as part of the same family in the earlier versions of PFAM. The functional relevance of such subfamily grouping in the current version needs to be tested further.

**Figure 5.**
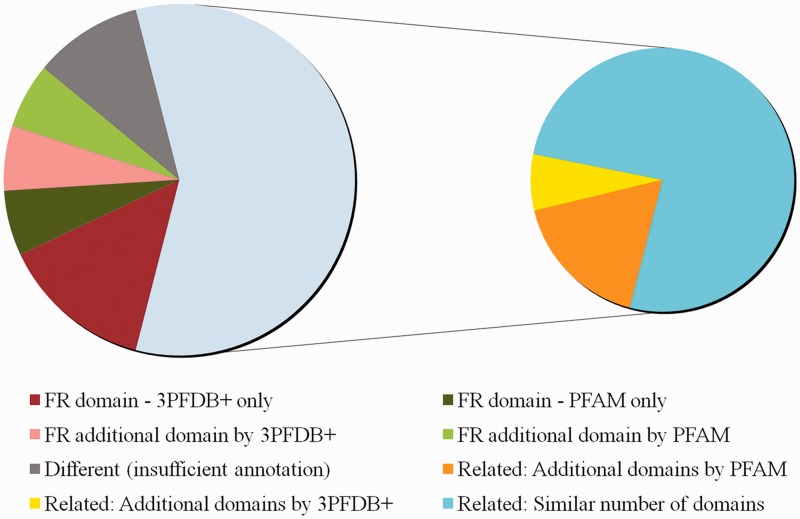
Comparison of domain assignments by PFAM and 3PFDB+ on 50 reviewed human proteins from UNIPROT. The distribution of sequences under different categories based on clear functional relevance of domain assignments is presented. Functionally relevant is abbreviated as FR. Domain assignments related by PFAM Clan grouping or GO annotations, are marked as ‘Related’. Sequences for which domain assignments were given only by 3PFDB+ or PFAM are indicated as ‘3PFDB+ only’ or ‘PFAM only’. Additional FR domains assigned by 3PFDB+ or PFAM are marked as ‘FR additional domain by 3PFDB+’ or ‘FR additional domain by PFAM’. Assignments with a combination of related and additional domains are subgrouped under ‘Related’ depending on additional assignments by PFAM or 3PFDB+. Related assignments with same number of domains are indicated as ‘Related: Similar number of domains’. The domain assignments which needed further assessments due to insufficient or no annotation (UNIPROT or GO), are grouped under ‘Different (insufficient annotation)’.

When compared to PFAM, domain assignment by 3PFDB+ was functionally relevant (FR) for 10 sequences whereas PFAM search did not result in any assignment for seven sequences and different assignments were given for the rest. We consulted UNIPROT (18) sequence annotation and GO (19) molecular function to determine whether the domain assigned in relevant in the context of protein function. PFAM profile-based assignments were FR for six sequences but 3PFDB+ failed to give any assignment for three cases and different for the rest. For the other five sequences, functional relevance of domain assignments could not be established due to incomplete or no annotation. Figure 5 provides a summary of the assignment comparison and the list of assignments and associated remarks are given as Supplementary Table S2.

Separately, a more specific dataset of 39 human biologically important proteins, implicated in tumor-associated pathways, were used to study domain assignments. The domain architecture assigned for 7 out of 39 sequences were different between PFAM and 3PFDB+. In three cases, 3PFDB+ profiles were helpful in discriminating insignificant PFAM domain assignments (as per the Gathering threshold (GA) scores, Supplementary Table S3). Insignificant matches could be due to dilution of family HMMs, owing to high sequence divergence in entire family alignments. For two sequences, 3PFDB+ assigns more domains which are relevant in the functional context of the protein and the architectures are found in other proteins in PFAM database. For two sequences, 3PFDB+ and PFAM assigns different but related domain families with same function.

## Discussion

The revised protocol for search for best representatives relies on an initial sequence search, starting from a set of family members selected at a 25% identity cut-off, for homologs using mere three iterations of PSI-BLAST. This search is carried out against a dataset obtained by a rather stringent filter of redundant entries at a 50% cut-off of sequence identity. This revised protocol ensures sufficient sampling of the sequence space, using multiple start points, without compensating much on the computational time. The best representatives identified in 3PFDB+, retained better family coverage when compared to the representatives identified earlier (6). Profiles weak in sequence data were enriched with sequences from the full family sets. This improved the quality of many of low coverage representatives.

### Partial domains and fragments

For 153 families, where the best representatives had coverage <90%, it is expected that these family members may form multiple subgroups and a single representative is not able to associate all these subgroups. In such cases, the use of more than one representative may be essential in providing a better family coverage.

**Figure 6.**
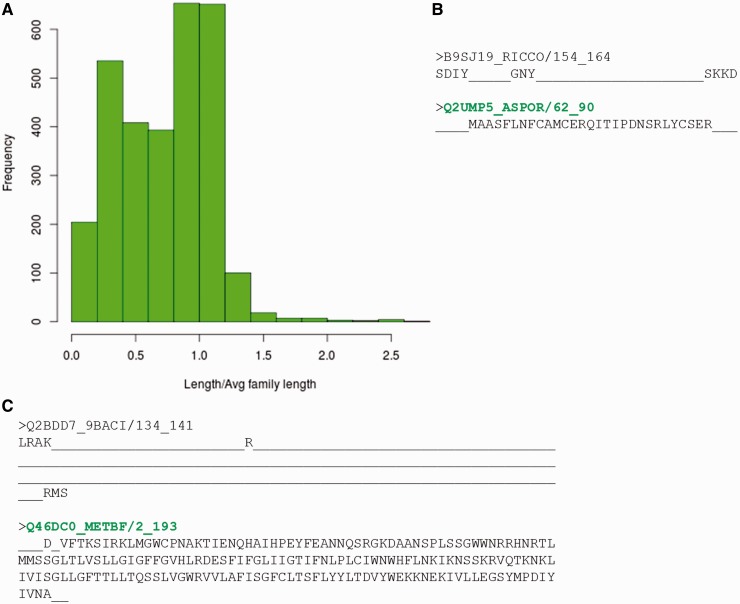
Sequences which do not recognize their family representatives. (**A**) Distribution of ratio of length of the sequence not identified by BRPs and average family length. (**B**, **C**) Alignment of the sequence not identified by BRP with the sequence of best representative. The PFAM family names are indicated in red and the BRS for these families are highlighted in green.

However, before assessing the possibility of multiple representatives, the reasons for non-identification of any of the family member by its representative profile, were analysed in detail. Sequences belonging to different PFAM families that fail to recognize the BRPs identified using both methods were gathered. This accounts to 2986 sequences spanning 910 families. 38% of these sequences had length <60% of the average family length (Figure 6A). Hence, they could be considered as ‘partial domains’ or without complete domain sequence information. Figure 6B,C highlights two cases where certain family members are short fragments and have no clear association with the other family sequences. These short sequences falsely associate with parts of the family profile and do not hold any family-specific signatures or motifs.

The partial domains were mainly identified in the P-loop containing nucleoside triphosphate hydrolase superfamily (PFAM clan: CL0023), Actin-like ATPase superfamily (PFAM clan: CL0108), Ribonuclease H-like superfamily (PFAM clan: CL0219), RNA dependant RNA polymerase (PFAM clan: CL0027), Thiamine diphosphate-binding superfamily (PFAM clan: CL0254), Glycosyl transferase GT-A (PFAM clan: CL0110), Drug/Metabolite transporter superfamily (PFAM clan: CL0184) and Thioredoxin-like family (PFAM clan: CL0172).

### Biased amino acid composition

**Figure 7.**
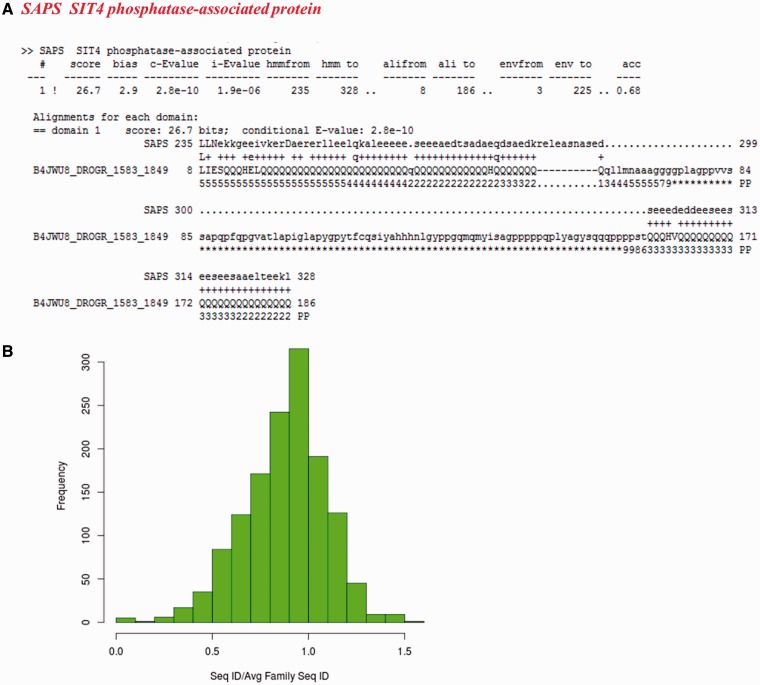
Sequences which do not recognize their family representatives and could not be categorized as partial domains or with multiple family associations. (**A**) Alignment of a low complexity sequence (identifier: B4JWU8_DROGR/1583-1849) with its family HMM. The family name is indicated in red. (**B**) Distribution of ratio of average sequence identity of the sequences not recognizing their BRPs (1383 in number) with other family members to the average family sequence identity calculated on the 50% non-redundant dataset.

For the other sequences not recognized as family members, we checked whether they have biased amino acid compositions. 205 of these non-fragment sequences have more than 30% of the residues in the regions of low complexity (Supplementary Table S4). Figure 7A presents one example from SIT4 phosphatase-associated protein family, where a poly-glutamate stretch is falsely associated with the family, whereas other members are not essentially glutamate-rich. 251 sequences exhibit compositional bias with 223 having transmembrane regions and 28 are rich in polar residues (more than 75%).

### Family outliers

A total of 1383 (46.3%) sequences do not belong to any of the above categories. These sequences are expected to be distant from the rest of the family members. They also lack one or more motifs that characterize the family, since FASSM was not able to recognize these sequences as part of the family. Ninety-nine per cent of these sequences are associated with families having representatives with coverage more than 90%.

**Figure 8.**
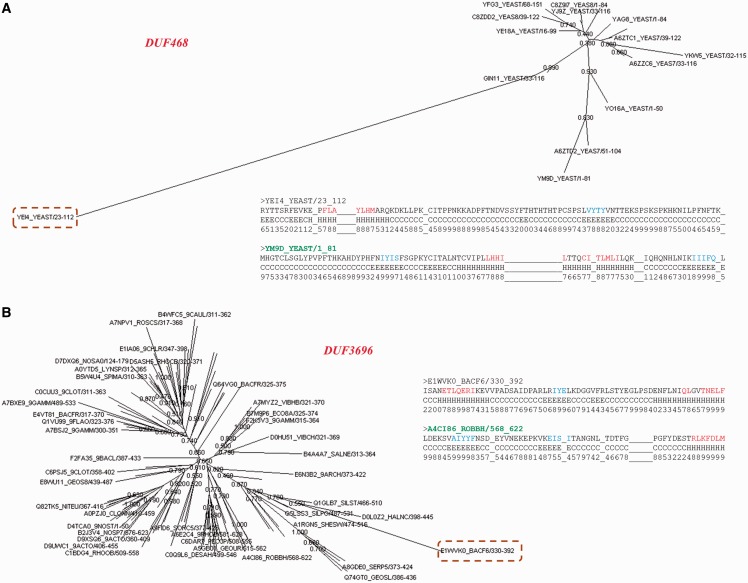
Family outliers. (**A**, **B**) Provision of two examples of sequences that could not be associated with family BRPs and are seen as outliers in the family. The association of this sequence with the other family members is presented using the Neighbor Joining tree obtained from PFAM database for the family. The outlier sequence is highlighted and the family name is given in red. Comparison of secondary structures predicted for the outlier and the BRS of the family are also presented as an alignment. The BRS is highlighted in green.

A total of 71.5% of these sequences are diverged with respect to other family members (Figure 7B). The ratio of average sequence identity obtained while comparing these sequences with other family members and the average family sequence identity is <1. 443 sequences have this ratio below 0.8 and they can be considered significantly far from the rest of the family. Many of these sequences are observed to be clear outliers in the family and secondary structure predictions suggest that the secondary structure topology of these sequences is quite different from other members (data not shown). Figure 8 shows two examples where a member is found to be an outlier and has been falsely or weakly associated with the family.

**Figure 9.**
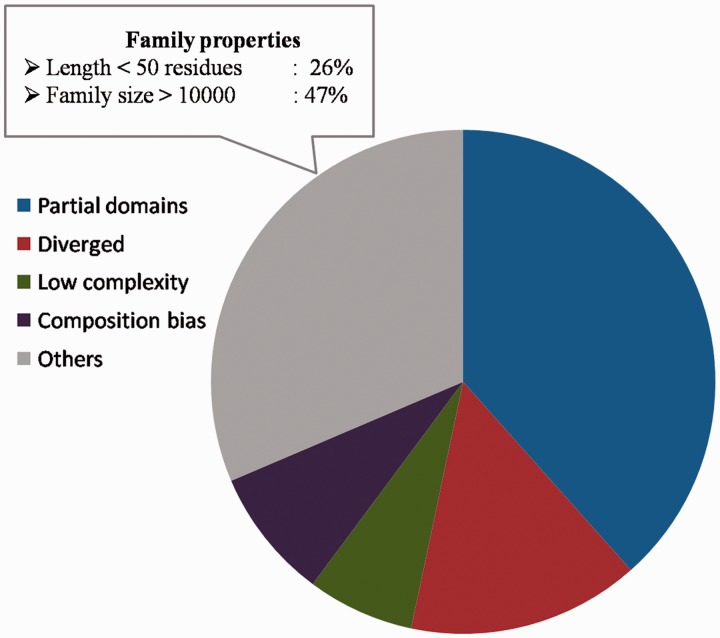
Sequences not recognized by representatives. The distribution of partial domains, low complexity sequences, compositionally biased sequences and diverged family members among those sequences not recognized as family members by the representatives. The family properties of those sequences not belonging to these categories are also highlighted.

Sequences for which the above reasons could not be attributed for not being recognized as part of the family, comprise 31.4% of the sequences (Figure 9) and they span 270 families. The family properties of these sequences were analysed. 244 sequences have average family sequence length <50 residues and 442 sequences have family size >10 000. Below the 50% sequence identity level, the average family sequence identity is <15% for 97% of the sequences as they belong to highly diverse PFAM families. The clans covering maximum number of these sequences include C2H2/C2HC zinc fingers, Tetratricopeptide repeats, OB fold, Ankyrin repeats and P-loop NTPases. Most of these clans are known for short and diverse sequences. The reliability of family association for these sequences need to be verified further and the representative profiles need to be enriched with more sequences for genuine cases of family memberships. The list of sequences for which we could associate reasons for not recognizing the family BRP can be downloaded from the database and Supplementary Table S5 lists the number of sequences and the reasons attributed for weak family association.

## Conclusions

BRSs were identified for 13 666 families in PFAM v 26 and corresponding representative profiles were also generated. New profile generation approach is adopted, which results in significant improvement in family coverage of the representatives. These representative sequences and profiles are presented in the 3PFDB+ database. Users can also search new sequences against the representative profiles using two efficient and sensitive sequence homology detection methods, HMMER (7) and FASSM (8).

Only 153 representatives have family coverage <90%. The low coverage is mainly associated with small families or short sequences. In-depth analysis of sequences that do not recognize their family BRPs helped to identify the reasons for weak family association. Nearly 40% of such sequences are partial domains or fragments that weakly or falsely associate with the family. Another major part corresponds to members that are either highly diverged from the family or are clear outliers that are falsely grouped into the family. A few sequences having low complexity regions were also found to have wrong family associations. Recognition of BRSs can reduce computational time for large-scale function annotation of gene products without compromising on coverage.

## Supplementary Data

Supplementary data are available at *Database* online.

## Funding

The authors thank NCBS (TIFR) for funding and infrastructural support. A.P.J. was supported by Human Frontier Science Program Grant of R.S. P.S. was supported by a scholarship by Department of Biotechnology, India. Funding for open access charge: NCBS.

*Conflict of interest*. None declared.
